# ELR+ CXC chemokine expression in benign and malignant colorectal conditions

**DOI:** 10.1186/1471-2407-8-178

**Published:** 2008-06-25

**Authors:** Claudia Rubie, Vilma Oliveira Frick, Mathias Wagner, Jochen Schuld, Stefan Gräber, Brigitte Brittner, Rainer M Bohle, Martin K Schilling

**Affiliations:** 1 Dept. of General -, Visceral-, Vascular – and Pediatric Surgery, University of the Saarland, 66421 Homburg/Saar, Germany; 2 Institute of Pathology, University of the Saarland, 66421 Homburg/Saar, Germany; 3 Institute of Medical Biometrics, Epidemiology, and Medical Informatics (IMBEI) University of the Saarland, 66421 Homburg/Saar, Germany

## Abstract

**Background:**

CXCR2 chemokine ligands CXCL1, CXCL5 and CXCL6 were shown to be involved in chemoattraction, inflammatory responses, tumor growth and angiogenesis. Here, we comparatively analyzed their expression profile in resection specimens from patients with colorectal adenoma (CRA) (n = 30) as well as colorectal carcinoma (CRC) (n = 48) and corresponding colorectal liver metastases (CRLM) (n = 16).

**Methods:**

Chemokine expression was assessed by microdissection, quantitative real-time PCR (Q-RT-PCR), the enzyme-linked immunosorbent assay (ELISA) and immunohistochemistry (IHC).

**Results:**

In contrast to CXCL6, we demonstrated CXCL1 and CXCL5 mRNA and protein expression to be significantly up-regulated in CRC and CRLM tissue specimens in relation to their matched tumor neighbor tissues. Moreover, both chemokine ligands were demonstrated to be significantly higher expressed in CRC tissues than in CRA tissues thus indicating a progressive increase in the transition from the premalignant condition to the development of the malignant status. Although a comparative analysis of the CXCL1/CXCL5 protein expression profiles in CRC patients revealed that the absolute expression level of CXCL1 was significantly higher in comparison to CXCL5, mRNA- and protein overexpression of CXCL5 in CRC and CRLM tissues was much more pronounced (80- and 60- fold in CRC tissues, respectively) in comparison to CXCL1 (5- and 3.5- fold in CRC tissues, respectively).

**Conclusion:**

Our results demonstrate a significant association between CXCL1 and CXCL5 expression with CRC and CRLM suggesting for both chemokine ligands a potential role in the progression from CRA to CRC and thus, in the initiation of CRC.

## Background

To date, colorectal cancer (CRC) constitutes one of the leading causes of cancer-related deaths worldwide. In spite of many currently available and continuously improving therapies for early stage colon cancer, the majority of CRC patients develop metastases which worsen the prognosis for survival dramatically [1,2].

The development of cancer metastases is a complex process consisting of various interacting mechanisms, which involve numerous biochemical and immunological changes like abnormally expressed growth factors and cytokines [3]. One group of cytokines that recently attracted a lot of attention with view to cancer-related mechanisms, are small signaling molecules termed chemokines [4]. Chemokines were originally identified in inflammatory pathways stimulating the migration of leucocytes, such as neutrophils and natural killer cells [5,6]. Only in recent years chemokines were reported to induce the migration of tumor cells and their expression was correlated with tumor growth, angiogenesis and metastatic potential in various human carcinomas and animal models [7-9].

In the present study, we monitored the expression profiles of several ELR+ CXC chemokines and their corresponding receptor CXCR2 in benign and malign colorectal conditions and in CRLM. We focused on CXCL1 [growth-related oncogene α (GROα)] [10], CXCL5 [epithelial neutrophil -activating protein 78 (ENA-78)] [11] and CXCL6 [granulocyte chemotactic protein-2 (GCP2)] [12], all structurally related members of the CXC chemokine family. This chemokine family is further subdivided into ELR^+ ^and ELR^- ^CXC chemokines, based on the presence or absence of the amino acid sequence Glu-Leu-Arg (ELR) preceding the first conserved cysteine amino acid residue in the primary structure of the CXC chemokines. All three chemokines under investigation contain the ELR motif, which is important for ligand/receptor interactions and the regulation of CXC chemokine induced angiogenesis [13-15].

The angiogenic activity of all ELR^+ ^CXC chemokines is mediated by the G-protein-coupled receptor CXCR2 [16], although some ELR^+ ^CXC chemokines also signal through CXCR1. While CXCL1 activates and interacts exclusively by CXCR2, CXCL5 and CXCL6 signal through both receptors [17].

All three genes are structurally related to another member of the ELR^+ ^chemokines, CXCL8 [interleukin-8 (IL-8)], which has recently been associated with CRC pathology and various other tumor types [18-21]. Like CXCL8 and other members of the ELR^+ ^CXC chemokine family, CXCL6 is a neutrophil chemoattractant that was recently demonstrated to promote tumor growth through its angiogenic effects in human tumors and animal models [22,23].

Involvement of CXCL5 has also been reported in different neoplastic processes with major focus on non small cell lung cancer (NSCLC), where CXCL5 was shown to be an important angiogenic factor [24]. Accordingly, in pancreatic cancer cell lines angiogenic activity was demonstrated [25] and with view to CRC elevated protein quantities have been reported [26].

The third ELR+ chemokine under investigation, CXCL1, originally identified as melanoma growth stimulating activity, has recently been adressed a role in HIV-infection [27] and correlated with tumorigenic and angiogenic effects and metastasis in squamous cell carcinoma and melanoma [28,29]. Recently, prostaglandin E_2 _(PGE_2_) was shown to induce the expression of CXCL1 in human CRC cells and to induce microvascular endothelial cell migration and tube formation in vitro [30]. Moreover, elevated CXCL1 expression has recently been associated with an invasive phenotype in human colon carcinoma cells [31] and down-regulation of the matrix protein fibulin-1 [32].

Despite the increasing number of studies indicating a role for CXCL1, CXCL5 and CXCL6 in different cancer types and processes, their relevance with view to the development of CRC is still rather limited. Thus, we were prompted to investigate their expression profiles in colorectal adenoma (CRA) specimens as premalignant stages in the development of CRC, further in CRC tissues of different tumor categories and corresponding colorectal liver metastases (CRLM), to track potential differences in the expression levels between the transition from a premalignant condition to a colorectal malignancy.

## Methods

### Patients

Surgical specimens and corresponding normal tissue from the same samples were collected from patients with CRA (n = 30), CRC of different tumor categories (n = 48) and synchronous or metachronous CRLM (n = 16) who underwent surgical resection at our department between January 2003 and october 2006. Informed consent for tissue procurement was obtained from all patients. The study was approved by the ethics commission of the Ärztekammer of the Saarland, Germany. The clinical variables presented in Table 1 and 2 were obtained from the clinical and pathological records and are in accordance with the UICC/TNM classification [33].

**Table 1:** Clinical characteristics of patients with colorectal carcinomas and colorectal liver metastases

Factor	CRC^2 ^*n *= 48	CRLM^3 ^*n *= 16^4^
Localization of primary tumor		
Colon	22	6
Rectum	26	8
Gender		
Male	29	7
Female	19	7
Age, yr^5^	63.7 (47–78)	60,3 (41–76)
Largest tumor diameter (cm)^5^	4.6 (1.2–9.1)	4,2 (1.5–5.5)
TNM^1 ^category of primary tumor		
I	8	1
II	15	2
III	15	10
IV	10	1
Grading		
I	0	0
II	21	4
III	27	10
Chemotherapy before operation	0	2
Radiotherapy before operation	0	2

^1^Tumor-node-metastasis; ^2^Colorectal carcinoma; ^3^Colorectal liver metastases; ^4^16 CRLM originating from 14 CRC patients; ^5^Median with range in parentheses.

**Table 2:** Clinical characteristics of patients with colorectal adenomas

Factor	CRA^1 ^*n *= 30
Localization of colorectal adenoma, respectively	
Colon	23
Rectum	7
Gender	
Male	17
Female	13
Age, yr^2^	65.7 (42–75)

^1^Colorectal adenoma; ^2^Median with range in parentheses.

### Tissue preparation

Tissue samples were collected immediately after resection, snap frozen in liquid nitrogen and then stored at -80°C until they were processed under nucleic acid sterile conditions for RNA and protein extraction. Tumor samples were taken from vital areas of histopathologically confirmed (R.M.B. and M.W.) adenocarcinomas and liver metastases, respectively. As corresponding normal tissue we used adjacent unaffected mucosa, 2–3 cm distal to the resection margin from the same resected adenocarcinoma or liver specimen, respectively. All tissues obtained were reviewed by a minimum of two experienced pathologists (M.W. *et al*.) and examined for the presence of tumor cells. As minimum criteria for usefulness for our studies we only chose tumor tissues in which tumor cells occupied a major component (> 80%) of the tumor sample.

### Single-strand cDNA synthesis

Total RNA was isolated using RNeasy columns from Qiagen (Qiagen, Hilden, Germany) according to the manufacturer's instructions. RNA integrity was confirmed spectrophotometrically and by electrophoresis on 1% agarose gels. For cDNA synthesis 5 μg of each patient total RNA sample were reverse-transcribed in a final reaction volume of 50 μl containing 1× TaqMan RT buffer, 2.5 μM/l random hexamers, 500 μM/l each dNTP, 5.5 mM/l MgCl_2_, 0.4 U/μl RNase inhibitor, and 1.25 U/μl Multiscribe RT. All RT-PCR reagents were purchased from Applied Biosystems (Applied Biosystems, Foster City, CA). The reaction conditions were 10 min at 25°C, 30 min at 48°C, and 5 min at 95°C.

### Real-time PCR

Expression profiles of CXCL1, CXCL5 and CXCL6 were monitored in CRA and CRC tissue specimens and corresponding CRLM by Q-RT-PCR. Adjacent, disease and tumor free colorectal epithelium and liver samples served as control groups, respectively. All Q-RT PCR assays containing the primer and probe mix were purchased from Applied Biosystems and utilized according to the manufacturer's instructions. PCR reactions were carried out using 10 μl 2× Taqman PCR Universal Master Mix No AmpErase^® ^UNG (Applied Biosystems) and 1 μL gene assay, 8 μL RNase-free water and 1 μl cDNA template (50 mg/l). The theoretical basis of the Q-RT assays is described in detail elsewhere [34]. All reactions were carried out in duplicate along with no template controls and an additional reaction in which reverse transcriptase was omitted to assure absence of genomic DNA contamination in each RNA sample. For signal detection, the ABI Prism 7900 sequence detector (Applied Biosystems), was programmed to an initial step of 10 min at 95°C, followed by 40 thermal cycles of 15 s at 95°C and 10 min at 60°C and the log-linear phase of amplification was monitored to obtain C_T _values for each RNA sample.

Gene expression of all target genes was analyzed in relation to the levels of the slope matched housekeeping genes phosphomannomutase (PMM1) and cyclophilin C (CycC) [35]. Since reporting of data obtained from raw C_T _values falsely represent the variations, we converted the individual C_T _values to the linear form as follows:

$$\text{Fold difference}=2^{-({\text{mean C}}_{\text{T}}\text{ pathological tissue}-{\text{mean C}}_{\text{T}}\text{ calibrator})}=2^{-{\text{delta C}}_{\text{T}}}$$

Consequently, the normal tissue becomes the 1 × sample, and all other quantities are expressed as an n-fold difference relative to this tissue.

### Isolation of total protein

Protein lysates from frozen tissues were extracted with a radioimmunoprecipitation (RIPA) cell lysis and extraction buffer (Pierce, Rockford, USA). Total protein content was assessed by using the Pierce BCA protein assay reagent kit (Pierce).

### Enzyme-linked immunosorbant assay (ELISA)

Tissue concentrations of CXCL1, CXCL5 and CXCL6 in the lysates were determined by sandwich-type ELISA according to the manufacturer's protocol: R&D systems (DuoSet, R&D Systems Inc. Minneapolis, Minnesota, USA). The absorbance was read at 450 nm.

### Immunohistochemistry

Operative specimens were routinely fixed in formalin and subsequently embedded in paraffin. Before staining, 4-μm thick paraffin-embedded tissue sections were mounted on Superfrost Plus slides, deparaffinized and rehydrated in graded ethanol to deionized water. The sections were microwaved with an antigen retrieval solution (Target Retrieval, Dakocytomation, Carpinteria, CA, USA) and after blocking of endogenous peroxidase activity with 3% hydrogen peroxide, the sections were further blocked for 30 minutes with normal rabbit serum. Overnight incubation at 4°C with primary goat polyclonal anti-human CXCL5 antibody (15 μg/ml, AF254, R&D Systems, Minneapolis, Minn., USA) or primary goat anti-human CXCL1 antibody (3.5 μg/ml, sc-1374, Santa Cruz Biotechnology, Santa Cruz, Calif., USA) was followed by incubation of secondary biotinylated biotinylated rabbit anti-goat IgG antibody and the avidin-biotin-peroxidase reaction (Vectastain ABC ELITE Kit, Vector Laboratories, Burlingame, CA, USA). After colour reaction with aminoethylcarbazol solution (Merck, Darmstadt, Germany), tissues were counterstained with haematoxylin. Negative controls were performed in all cases omitting primary antibody. All pictures were taken using a digital camera (Olympus DP71, Olympus Optical Co, Ltd, Tokyo, Japan).

### Laser Capture Microdissection

Laser microbeam microdissection (LMM) was employed for obtaining pure tumor cell and pure normal cell samples for subsequent genetic analysis. LMM was performed on three samples for each tissue type for CXCL1, CXCL5 and CXCL6. Histochemical staining was used on cryo sections before microdissection. Specimen preparation, microdissection and catapulting were performed following a laser pressure catapulting protocol according to the manufacturer's instructions (P.A.L.M. Microlaser Technologies, Bernried, Germany). RNA was extracted using the P.A.L.M. RNA extraction kit and for reverse transcription the invitrogen reverse transcription kit (Invitrogen Life Technologies, Karlsruhe, Germany) was applied. Subsequently quantitative PCR analysis was performed.

### Calculations and Statistical Methods

Expression profiles of CXCL1, CXCL5 and CXCL6 in the different groups are shown as mean and standard error of the mean (SEM). Statistical calculations were done with the MedCalc software package (MedCalc software, Mariakerke, Belgium) [36]. Where appropriate, either the Student's t-test or the Wilcoxon's rank sum test was applied to test for group differences of continuous variables. A *P *value of 0.05 or less was deemed significant. To measure how variables are related we performed a bivariate correlation analysis using the spearman ρ correlation coefficient.

## Results

### CXCL1, CXCL5 and CXCL6 mRNA expression

**Figure 1:**
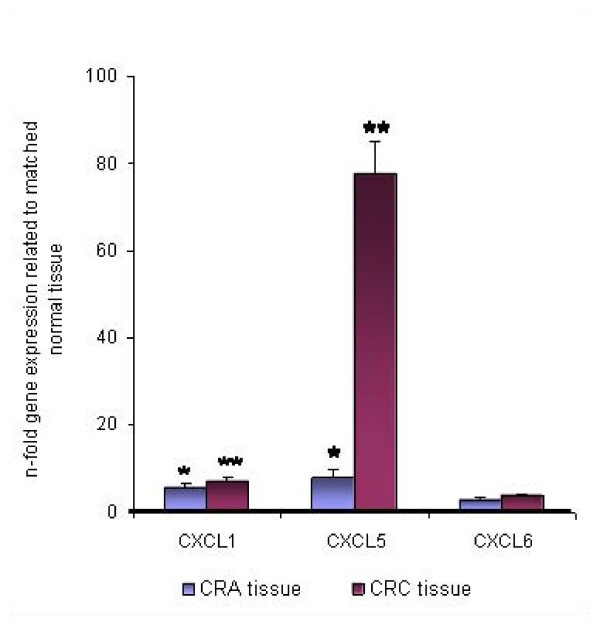
**Expression of CXCL1, CXCL5 and CXCL6 in colorectal adenoma (CRA) and colorectal carcinoma (CRC) tissue specimens as determined by Q-RT-PCR**. Q-RT-PCR data are expressed as mean +/- standard error of the mean (SEM), * *P *< 0.05, ** *P *< 0.001, *n *= 30 and 48, respectively. Fold increase above 1 indicates chemokine overexpression in CRA and CRC tissues related to unaffected neighbor tissues, respectively.

**Figure 2:**
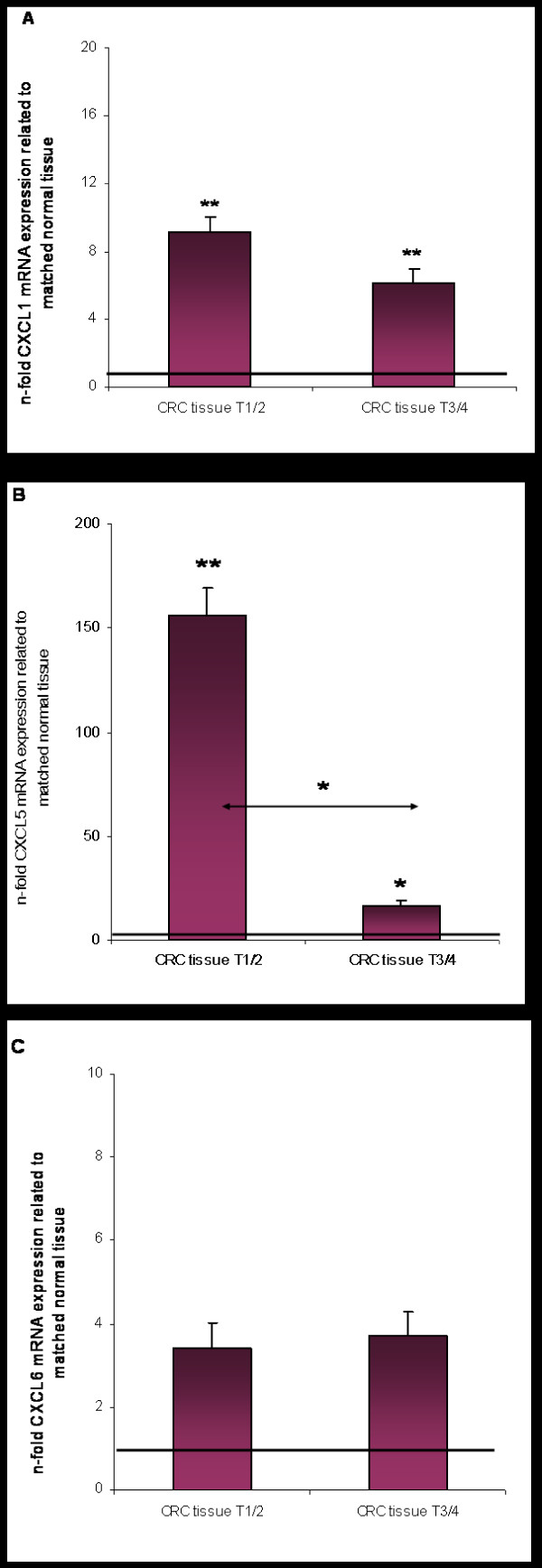
**Expression of (A) CXCL1, (B) CXCL5 and (C) CXCL6 in different tumor categories of colorectal carcinoma (CRC) as determined by Q-RT-PCR**. Q-RT-PCR data are expressed as mean +/- standard error of the mean (SEM), * *P *< 0.05, ** *P *< 0.001, *n *= 23 and 25, respectively. Fold increase above 1 indicates chemokine overexpression in CRC tissues related to unaffected neighbor tissues, respectively. Note the different scale for CXCL1, CXCL5 and CXCL6 expression.

**Figure 3:**
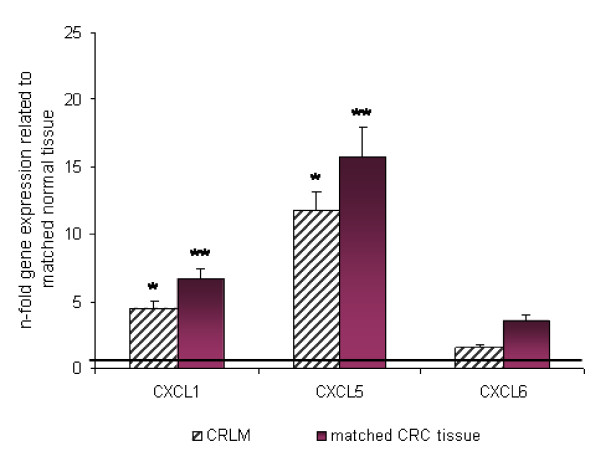
**Expression of CXCL1, CXCL5 and CXCL6 in colorectal liver metastases (CRLM) and corresponding primary colorectal tumors as determined by Q-RT-PCR**. Q-RT-PCR data are expressed as mean +/- standard error of the mean (SEM), * *P *< 0.05, ** *P *< 0.001, *n *= 16 and 14, respectively. Fold increase above 1 indicates chemokine overexpression in affected tissues related to unaffected neighbor tissues, respectively.

CXCL1 and CXCL5 mRNA expressions were significantly up-regulated in all CRC tissue specimens in relation to the matched tumor neighbor tissues (*P *< 0.001, respectively; Fig. 1). In addition, CXCL1 also revealed significant up-regulation in the CRA tissues (*P *< 0.05). In contrast, no significant up-regulation of CXCL6 mRNA expression was observed in either tissue type (Fig. 1). In CRA tissues also CXCL5 showed no significant up-regulation, indicating that CXCL5 is not overexpressed in preliminary stages of CRC. However, CXCL5 overexpression in CRC tissues revealed a much more pronounced 80-fold up-regulation in comparison to CXCL1, which only showed a 5-fold overexpression in CRC patients. Furthermore, we detected a significant clinicopathological association between CXCL5 expression and early tumor categories of CRC (*P *< 0.001), which did not occur for CXCL1 or CXCL6 (Fig. 2). In metastatic samples, CXCL1 and CXCL5 expression was also significantly elevated relative to corresponding normal liver tissues (*P *< 0.05, respectively, Fig. 3). Again, CXCL5 expression was much more pronounced. Between metastases and corresponding primary tumors we observed no significant difference in mRNA expression (Fig. 3). Notably, CXCL5 expression was significantly lower in the corresponding primary tumors (n = 14) of the CRLM presented in Fig. 3 compared to the entire CRC patient cohort (n = 48), which comprised all tumor categories 1–4 as shown in Fig. 1. Showing that lower T- categories (T1–2) express almost 100-fold significantly more CXCL5 than higher T-categories (T3–4) (Fig. 2B), led us to review the T- categories of the corresponding primary tumors of the CRLM. It turned out that 11 out of 14 tumors were classified as T3 and T4 tumors (Table 1), thus explaining the results presented in Fig. 2B.

To verify that up-regulation of CXCL1 and CXCL5 is generated exclusively by tumor cells rather than inflammatory cells or other non-malignant cells in the tissue blocks, we routinely analysed Q-RT-PCR expressions of several sections of microdissected tumor and normal cells from three specimens of all tissue types under investigation. Moreover, we generally determined the differences between gene expressions from matched normal/cancer samples constituting the basis for all our calculations.

### CXCL1, CXCL5 and CXCL6 protein expression

**Figure 4:**
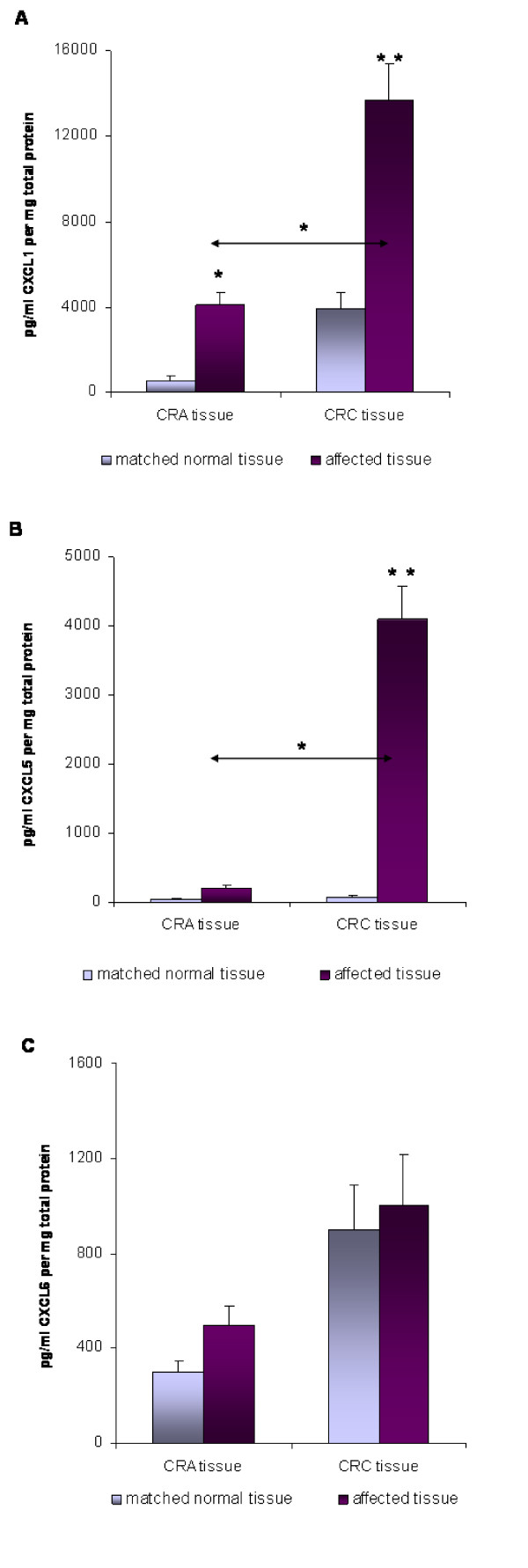
**Expression of (A) CXCL1, (B) CXCL5 and (C) CXCL6 in colorectal adenoma (CRA) and colorectal carcinoma (CRC) tissue specimens as determined by ELISA assays**. Elisa results are presented as absolute values of pg per ml chemokine ligand per mg total protein in CRA and CRC tissues and unaffected neighbor tissues, respectively. The data are expressed as mean +/- standard error of the mean (SEM), * *P *< 0.05, ** *P *< 0.001, *n *= 30 and 48, respectively. Note the different scale for CXCL1, CXCL5 and CXCL6 expression.

CXCL1 and CXCL5 protein expressions, as assessed by ELISA, showed a significant up-regulation in the CRC tissue specimens in comparison to the unaffected corresponding mucosa tissues, respectively (*P *< 0.001) as demonstrated in Figures 4A and 4B. In consistence with the results obtained at the mRNA level, CXCL5 protein overexpression was much more pronounced corresponding to a 60-fold expression increase compared to only a 3.5-fold expression increase for CXCL1. Although significant up-regulation of CXCL1 proteins was also detected in CRA tissues, the level of CXCL1 protein expression in the CRC tissues was significantly 3.5-fold higher than in the CRA tissues (*P *< 0.05, Fig. 4A). Similarly, CXCL5 showed significant protein up-regulation in the CRC patient tissues with respect to the CRA tissues (Fig. 4B). Again, this overexpression was much more pronounced in comparison to CXCL1, demonstrating a 20-fold up-regulation for CXCL5 versus a 3.5-fold up-regulation for CXCL1, thus indicating a prominent progressive protein expression increase in the transition from the premalignant condition to the development of colorectal malignancies. In contrast, CXCL6 protein expressions showed no significant up-regulation in either tissue type (Fig. 4C).

**Figure 5:**
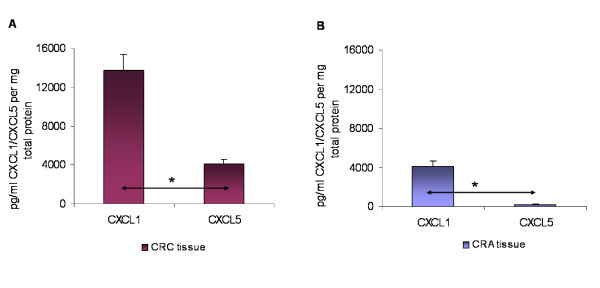
**Expression of CXCL1 and CXCL5 and in (A) colorectal carcinoma (CRC) and (B) colorectal adenoma (CRA) tissue specimens as determined by ELISA assays**. Elisa results are presented as absolute values of pg per ml chemokine ligand per mg total protein in CRC and CRA tissues and unaffected neighbor tissues, respectively. The data are expressed as mean +/- standard error of the mean (SEM), * *P *< 0.05, ** *P *< 0.001, *n *= 48 and 30, respectively.

Comparative analysis of the CXCL1/CXCL5 absolute protein quantities in CRC patients revealed a significantly higher CXCL1 expression (*P *< 0.05) of almost 14000 pg/ml compared to approximately 4000 pg/ml CXCL5 (Fig. 5A). On the other hand, we also observed a significantly higher CXCL1 expression level in the CRA tissues, where 4000 pg/ml CXCL1 compared to 200 pg/ml CXCL5, indicating that the basic expression level of CXCL1 is generally higher in both disease entities in comparison to CXCL5 (Fig. 5B).

### Cell-specific expression of CXCL1 and CXCL5 by IHC

**Figure 6:**
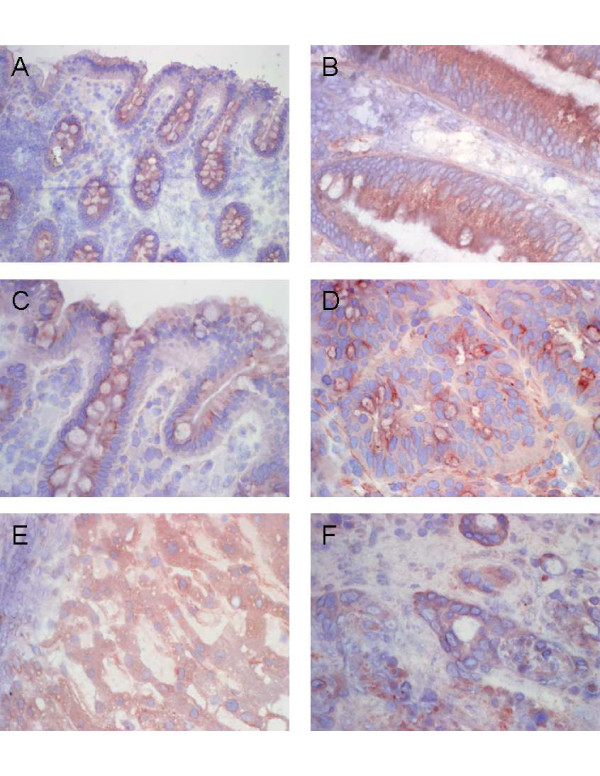
**Detection of CXCL1 protein expression in representative CRA, CRC and CRLM specimens as assessed by immunohistochemical staining with CXCL1 -specific antibodies**. (A) Weak immunostaining in apical epithelial cells within tumor neighbor tissues of CRA, (B) weak to medium staining intensities in apical epithelial cells within CRA specimens, (C) weak insular signals in apical epithelial cells and goblet cells within tumor neighbor tissues of CRC, (D) intense ubiquitous epithelial signals and insular mesenchymal signals in CRC tissues, (E) medium staining intensities of fine granular texture in hepatocytes and (F) intense cytoplasmic signals in ubiquitous distribution in CRLM (original magnification 200 and 400 fold, respectively).

**Figure 7:**
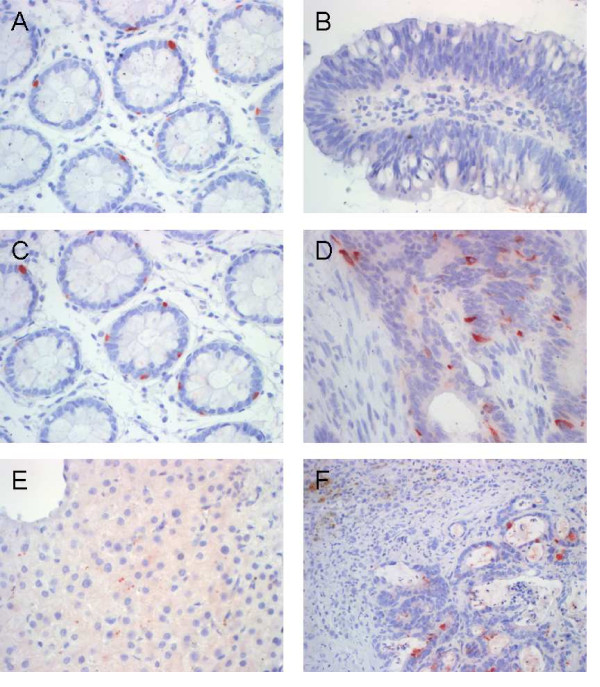
**Detection of CXCL5 protein expression in representative CRA, CRC and CRLM specimens as assessed by immunohistochemical staining with CXCL5 -specific antibodies**. (A) Weak cytoplasmic signals in basal crypt cells within tumor neighbor tissues of CRA, (B) very weak immunostaining in CRA specimens, (C) weak insular epithelial signals in tumor neighbor tissues of CRC, (D) intense basal and apical epithelial signals and interspersed rare mesenchymal signals in CRC tissues, (E) no substantial reactivity in corresponding neighbour tissues of CRLM and (F) intensive staining signals in epithelial and mesenchymal cells within tumor areas of CRLM specimens (original magnification 200 and 400 fold, respectively).

Detection of CXCL1 and CXCL5 expression was assessed by immunohistochemical staining in CRA, CRC and CRLM specimens and corresponding normal tissues (Fig 6). Immunostaining with CXCL1 -specific antibodies showed weak apical epithelial signals and weak staining of goblet cells in the corresponding normal areas of CRA and CRC tissues (Fig. 6A and 6C). Also in CRA tissues CXCL1 revealed only apical weak to medium staining of epithelial cells (Fig. 6B). In contrast, intense apical and basal CXCL1 staining signals were found in epithelial cells of CRC tissues and to a lesser extent also positive signals were observed in mesenchymal cells of the CRC tissues (Fig. 6D). In tumor neighbor tissues of CRLM we observed medium CXCL1 staining intensities of fine granular texture in hepatocytes (Fig. 6E) while Glisson's triangles were strictly negative (left upper corner of Fig. 6E). In contrast, CRLM specimens displayed intense cytoplasmic CXCL1 staining signals in ubiquitous distribution (Fig 6F). Interestingly, both colorectal tumor tissues and hepatic metastatic tumor tissues showed apical and basal distribution of CXCL1 signals, whereas CRA tissues and unaffected corresponding neighbor tissues of CRC and CRA displayed only apical CXCL1 signals. Also for CXCL5 we detected only very weak staining intensities in the unaffected corresponding neighbor tissues of CRC and CRA restricted to some insular cytoplasmic signals in basal crypt cells (Fig.7A and 7C). Likewise, no substantial immunostaining was detected within CRA tissue specimens for CXCL5 with the exception of very insular positive signals in mesenchymal or goblet cells (Fig. 7B). In contrast, we observed intense CXCL5 staining within CRC specimens mainly concentrated in epithelial cells and only randomly interspersed positive signals in mesenchymal cells (Fig. 7D). These findings indicate that the high CXCL5 expression detected in our mRNA and protein analysis is attributed to positive signals of tumor cells rather than infiltrating immune cells.

In tumor neighbor tissues of CRLM we detected no substantial CXCL5 reactivity. Only insular dotlike, cytoplasmic signals were randomly interspersed in some hepatocytes (Fig. 7E). In contrast, we observed intensive staining signals in epithelial and mesenchymal cells within tumor areas of CRLM specimens (Fig. 7F). Well confined from the positive signals in the tumor areas we also observed unspecific staining of bile pigments (left upper image border in Fig. 7F).

### Comparative CXCR2 expression in different colorectal conditions

**Figure 8:**
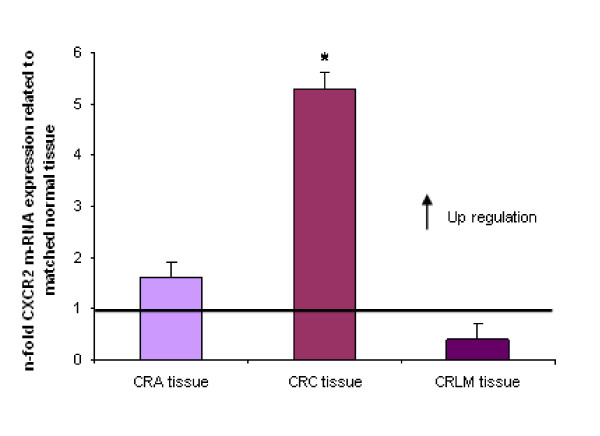
**Expression of CXCR2 in CRA, CRC and CRLM tissue specimens as determined by Q-RT-PCR**. Q-RT-PCR data are expressed as mean +/- standard error of the mean (SEM), * *P *< 0.05, ** *P *< 0.001, *n *= 30 (CRA), 48 (CRC) and 16 (CRLM), respectively. Fold increase above 1 indicates CXCR2 overexpression in CRA, CRC and CRLM tissues related to unaffected neighbor tissues, respectively.

While we observed no significant up- or down regulation of CXCR2 mRNA expression in CRA and CRLM tissue specimens, we found significant CXCR2 up-regulation in CRC tissues (*P *< 0.05) (Fig. 8).

## Discussion

This study provides evidence that significantly increased CXCL1 and CXCL5 expression associates with the transition from a premalignant condition to colorectal malignancies. In contrast, CXCL6 showed no association with colorectal malignancies in our studies. CXCR2, the corresponding receptor, respectively, showed also significant up-regulation in CRC tissues and no significant up-regulation in the CRA tissues thus matching the expression profile of CXCL1 and CXCL5.

As we have shown previously (21) CXCL8 mRNA and protein expression is significantly up-regulated in CRC specimens in relation to CRA and UC tissues. Moreover, CXCL8 expression revealed a close correlation with tumor grading. Most interestingly, CXCL8 up-regulation was most enhanced in synchronous and metachronous CRLM, if compared with the corresponding primary CRC tissues thus suggesting an association between CXCL8 expression, induction and progression of colorectal carcinoma and the development of colorectal liver metastases. Thus, we observed for the expression levels of CXCL8, CXCL1 and CXCL5 a significant association with the development of colorectal carcinoma and also CRLM. For CXCL8 we also observed on the protein level that the expression was significantly higher in tumor stage 4 in comparison to the lower tumor stages. Also Q-RT-analysis revealed a similar effect for CXCL8.

However, in recent years a number of studies have emerged demonstrating CXCL6 involvement in tumor development and angiogenesis in various animal models and human tumors [22,23]. Thus, van Coillie et al provided evidence that mouse CXCL6 gene transfer enhanced the development of Bowes melanoma tumors coupled with a higher neutrophil influx and significantly increased angiogenesis [22]. Moreover, CXCL6 production by endothelial cells within gastrointestinal tumors was shown to contribute to tumor development and neovascularization [23] and constitutive CXCL6 secretion was recently demonstrated in a panel of small cell lung cancer (SCLC) cell lines and in clinical SCLC specimens [37].

Despite the increasing number of studies suggesting a cancer-related role for CXCL6, we observed no significant CXCL6 up-regulation in CRC tissues or CRLM, neither on the RNA nor on the protein level. Moreover, we measured no significant difference in CXCL6 expression between CRA, CRC or CRLM tissues in our patient cohort. In contrast, both other chemokines under investigation, CXCL1 and CXCL5, showed significant RNA and protein up-regulation in all colorectal malignancies comprising CRC and CRLM tissues. Accordingly, our IHC results confirmed that this up-regulation resulted from tumor cells as we detected intense CXCL1 and CXCL5 staining signals in CRC and CRLM specimens mainly concentrated in the epithelial cells of these tissues.

These findings are supported by a number of recent studies adressing a cancer-related role to these chemokines.

Thus, elevated CXCL5 protein levels have been reported in CRC patients [26] and in human pancreatic cancer cell lines [25]. The mechanistic involvement of CXCL5 in the promotion of NSCLC was recently investigated in a study by Pöld et al [38], who demonstrated that enhanced tumor growth of COX-2 over-expressing tumors in a immunodeficient mouse model was associated with enhanced CXCL5 expression and inhibited by neutralizing anti-CXCL5 sera.

Also CXCL1 has recently been shown to promote tumor growth and angiogenesis in different tumor types. In human CRC cells, Wang et al [30] demonstrated recently, that PGE_2 _induced the expression of CXCL1 in the human CRC cells and also microvascular endothelial cell migration and tube formation in vitro. Moreover, the authors observed that PGE_2 _promoted tumor growth in vivo by induction of CXCL1 expression resulting in increased tumor microvessel formation. In murine models of squamous cell carcinoma and Lewis lung cancer CXCL1 expression was shown to parallel tumor growth [28,39] and in adrenocortical and prostate carcinoma [40,41] as well as in melanoma [29,42,43] CXCL1 was shown to mediate tumorigenicity. Evidence supporting a role for CXCL1 in CRC pathology was recently provided by a study demonstrating an association between increased expression of CXCL1 and down-regulation of the matrix protein fibulin-1, which is known to suppress the motility of various cancer cells [32]. Accordingly, Li et al [31] investigated CXCL1 expression in human colon carcinoma cells with different metastatic potentials and demonstrated that constitutive expression of CXCL1 was associated with metastatic potential and modulation of colon cancer cell proliferation [31].

In line with these findings we also observed significant up-regulation of CXCL1 in neoplastic colorectal tissues. Moreover, we found significantly elevated CXCL1 expression in the CRA tissues of our patient cohort. However, both chemokines CXCL1 and CXCL5 were shown to be significantly higher expressed in the CRC tissues in comparison to the CRA tissues. Since CRA constitutes a premalignant condition often preceding the development of colorectal malignancies, we hypothesized that a significant increase of chemokine expression in the CRC tissues with respect to the CRA tissues may indicate a transition from the premalignant condition to the development of the malignancy. This conclusion is supported by previous studies showing elevated GROa expression in the intestinal mucosa of patients with ulcerative colitis and Crohn's disease which are known risk factors for the development of CRC [44,45]. Although the basic expression level of CXCL1 was generally higher in both disease entities in comparison to CXCL5, we detected that CXCL5 overexpression was significantly more pronounced compared to CXCL1. Moreover, we observed a significant clinicopathological association between CXCL5 expression and early tumor categories of CRC which matched the expression status of the corresponding primary tumors of the CRLM in our investigation.

## Conclusion

In our study, we outlined a prominent expression profile for both chemokines, CXCL1 and CXCL5 with respect to CRC pathology. On the basis of the current literature CXCL1 appears to have a role in CRC related malignancies. However, CXCL5 exhibited a much more pronounced significant overexpression in the CRC and CRLM tissues with respect to the CRA tissues thus indicating a vast progressive increase in the transition from the premalignant condition to the development of the malignant status and thus, in the initiation of CRC. Since our results are based merely on descriptive expression data, future functional tests will be needed for further evaluation of the precise pathophysiological role. However, the CXCL1 and CXCL5 expression profiles outlined in this study strongly recommend monitoring the CXCL1 and CXCL5 expression in patients with CRA and CRC. Thus, we suggest that targeting CXCL1 and CXCL5 signaling may be a useful future tool in CRC pathology.

## Competing interests

The authors declare that they have no competing interests.

## Authors' contributions

All authors read and approved the final manuscript.

CR is responsible for the design of the study, interpretation of the results and drafted the manuscript. VOF took part in all experimental elements, performed the ELISAs and IHCs and participated in scientific discussions and interpretation of the results. MW examined the tissue sections for the presence of tumor cells, histopathologically confirmed all tissues under investigation, participated in scientific discussions and data interpretation. JS contributed to the collection of tissue specimens and provided clinical information. SG participated in the statistical analysis. RMB contributed to the histopathological survey and participated in scientific discussions. MKS is responsible for the provision of all the patient material and clinical information, participated in scientific discussions, data interpretation and revision of the manuscript.

## Pre-publication history

The pre-publication history for this paper can be accessed here:



## References

[B1] Booth RA (2007). Minimally invasive biomarkers for detection and staging of colorectal cancer. Cancer Lett.

[B2] McLeod HL, McKay JA, Collie-Duguid ES, Cassidy J (2000). Therapeutic opportunities from tumor biology in metastatic colon cancer. Eur J Cancer.

[B3] Nicolson GL (1991). Tumor and host properties and the role of oncogenes and suppressor genes. Curr Opin Oncol.

[B4] Baggiolini M (2001). Chemokines in pathology and medicine. J Intern Med.

[B5] Luster AD (1998). Chemokines-chemotactic cytokines that mediate inflammation. N Engl J Med.

[B6] Zlotnik A, Yoshie O (2000). Chemokines: a new classification system and their role in immunity. Immunity.

[B7] Wang JM, Deng X, Gong W, Su S (1998). Chemokines and their role in tumor growth and metastasis. J Immunol Methods.

[B8] Rubie C, Oliveira V, Kempf K, Wagner M, Tilton B, Rau B, Kruse B, Konig J, Schilling M (2006). Involvement of chemokine receptor CCR6 in colorectal cancer metastasis. Tumour Biol.

[B9] Rubie C, Frick VO, Wagner M, Rau B, Weber C, Kruse B, Kempf K, Tilton J, König J, Schilling M (2006). Enhanced expression and clinical significance of CC-chemokine MIP-3alpha in hepatocellular carcinoma. Scan J Immunol.

[B10] Sager R, Anisowicz A, Pike MC, Beckmann P, Smith T (1992). Structural, regulatory, and functional studies of the GRO gene and protein. Cytokines.

[B11] Walz A, Burgener R, Car B, Baggiolini M, Kunkel SL, Strieter RM (1991). Structure and neutrophil-activating properties of a novel inflammatory peptide (CXCL5) with homology to interleukin-8. J Exp Med.

[B12] Rovai LE, Herschman HR, Smith JB (1997). Cloning and characterization of the human granulocyte chemotactic protein-2 gene. J Immunol.

[B13] Strieter RM, Polverini PJ, Kunkel SL, Arenberg DA, Burdick MD, Kasper J, Dzuiba J, Van Damme J, Walz A, Marriott D, Chan SY, Roczniak S, Shanafelt AB (1995). The functional role of the ELR motif in CXC chemokine-mediated angiogenesis. J Biol Chem.

[B14] Moore BB, Arenberg DA, Addison CL, Keane MP, Polverini PJ, Strieter RM (1998). CXC chemokines mechanism of action in regulating tumor angiogenesis. Angiogenesis.

[B15] Strieter RM, Burdick MD, Mestas J, Gomperts B, Keane MP, Belperio JA (2006). Cancer CXC chemokine networks and tumour angiogenesis. Eur J Cancer.

[B16] Keane MP, Burdick MD, Xue YY, Lutz M, Belperio JA, Strieter RM (2004). The chemokine receptor CXCR2 mediates the tumorigenic effects of ELR+ CXC chemokines. Chest.

[B17] Addison CL, Daniel TO, Burdick MD, Liu H, Ehlert JE, Xue YY, Buechi L, Walz A, Rrichmond A, Strieter RM (2000). The CXC cemokine receptor, CXCR2, is the putative receptor for ELR+ CXC chemokine-induced angiogeneic activity. J Immunol.

[B18] Li A, Varney ML, Singh RK (2001). Expression of interleukin 8 and its receptors in human colon carcinoma cells with different metastatic potentials. Clin Cancer Res.

[B19] Brew R, Erikson JS, West DC, Kinsella AR, Slavin J, Christmas SE (2000). Interleukin-8 as an autocrine growth factor for human colon carcinoma cells *in vitro*. Cytokine.

[B20] Xie K (2001). Interleukin-8 and human cancer biology. Cytokine Growth Factor Rev.

[B21] Rubie C, Frick VO, Pfeil S, Wagner M, Kollmar O, Kopp B, Gräber S, Rau BM, Schilling MK (2007). Correlation of CXCL8 with induction, progression and metastatic potential of colorectal cancer. World J Gastroenterol.

[B22] Van Coillie E, Aelst IV, Wuyts A, Vercauteren R, Devos R, De Wolf-Peeters C, Van Damme J, Opdenakker G (2001). Tumor angiogenesis induced by granulocyte chemotactic protein-2 as a countercurrent principle. Am J Pathol.

[B23] Gijsbers K, Gouwy M, Struyf S, Wuyts A, Proost P, Opdenakker G, Penninckx F, Ectors N, Geboes K, Van Damme J (2005). GCP-2/CXCL6 synergizes with other endothelial cell-derived chemokines in neutrophil mobilization and is associated with angiogenesis in gastrointestinal tumors. Exp Cell Res.

[B24] Arenberg DA, Keane MP, DiGiovine B, Kunkel SL, Morris SB, Xue YY, Burdick MD, Glass MC, Iannettoni, Strieter RM (1998). Epithelial-neutrophil activating peptide (CXCL5) is an important angiogenic factor in non-small cell lung cancer. J Clin Invest.

[B25] Wente MN, Keane MP, Burdick MD, Friess H, Büchler MW, Ceyhan GO, Reber HA, Strieter RM, Hines OJ (2006). Blockade of the chemokine receptor CXCR2 inhibits pancreatic cancer cell-induced angiogenesis. Cancer Lett.

[B26] Baier PK, Eggstein S, Wolff-Vorbeck G, Baumgartner U, Hopt UT (2005). Chemokines in human colorectal carcinoma. Anticancer Res.

[B27] Lane BR, Strieter RM, Coffey MJ, Markovitz DM (2001). Human immunodefiency virus type 1 (HIV-1)-induced GRO-α production stimulates HIV-1 replication in macrophages and T lymphocytes. J Virol.

[B28] Loukinova E, Dong G, Enamorado-Ayalya I, Thoma GR, Chen Z, Schreiber H, Waes CV (2000). Growth-regulated oncogene-α expression by murine squamous cell carcinoma promotes tumor growth, metastasis, leukocyte infiltration and angiogenesis by a host CXC receptor-2 dependent mechanism. Oncogene.

[B29] Luan JR, Shattuk-Brandt R, Haghnegahdar H, Owen JD, Strieter R, Burdick M, Nirodi C, Beauchamp D, Johnson KN, Richmond A (1997). Mechanism and biological significance of constitutive expression of MGSA/GRO chemokines in malignant melanoma tumor progression. J Leucocyte Biol.

[B30] Wang D, Wang H, Brown J, Daikoku T, Ning W, Shi Q, Richmond A, Strieter R, Dey S, DuBois R (2006). CXCL1 induced by PGE2 promotes angiogenesis in colorectal cancer. J Exp Med.

[B31] Li A, Varney ML, Singh RK (2004). Constitutive expression of growth regulated oncogene (gro) in human colon carcinoma cells with different metastatic potential and its role in regulating their metastatc phenotype. Clin Exp Metastasis.

[B32] Wen Y, Giardina SF, Hamming D, Greenman J, Zachariah E, Bacolod MD, Liu H, Shia J, Amenta PS, Barany F, Paty P, Gerald W, Notteermann D (2006). CXCL1 is highly expressed in adenocarcinoma of the colon and down-regulates fibulin-1. Clin Cancer Res.

[B33] Sobin LH, Fleming ID (1997). TNM classification of malignant tumors, fifth edition (1997). Union Internationale Contre le Cancer and the American Joint Committee on Cancer. Cancer.

[B34] Bustin SA (2000). Absolute quantification of mRNA using real time reverse transcription polymerase chain reaction assays. J Mol Endocrinol.

[B35] Rubie C, Kempf K, Hans J, Su T, Tilton B, Georg T, Brittner B, Ludwig B, Schilling M (2005). Housekeeping gene variability in normal and cancerous colorectal, pancreatic, esophageal, gastric and hepatic tissues. Mol Cell Probe.

[B36] Schoonjans F, Zalata A, Depuydt CE, Comhaire FH (1995). MedCalc: a new computer program for medical statistics. Comput Methods Programs Biomed.

[B37] Zhu YM, Bagstaff SM, Woll PJ (2006). Production and up-regulation of granulocyte chemotactic protein-2/CXCL6 by IL-Iβ and hypoxia in small cell lung cancer. Brit J Cancer.

[B38] Pöld M, Zhu LX, Sharma S, Murdick M, Lin Y, Lee P, Pöld A, Luo J, Krysan K, Dohadwala M, Mao JT, Batrea RK, Strieter RM, Dubinett SM (2004). Cyclooxygenase-2-dependent expression of angiogenic CXC chemokines CXCL5/CXC ligand (CXCL) 5 and interleukin-8/CXCL8 in Human Non-Small cell lung cancer. Cancer Res.

[B39] Keane MP, Belperio JA, Xue YY, Burdick MD, Strieter RM (2004). Depletion of CXCR2 inhibits tumor growth and angiogenesis in a murine model of lung cancer. J Immunol.

[B40] Schteingart DE, Giordano TJ, Benitez RS, Burdick MD, Starkman MN, Arenberg DA, Strieter RM (2001). Overexpression of CXC chemokines by an adrenocortical carcinoma: a novel clinical syndrome. J Clin Endocrinol Metab.

[B41] Moore BB, Arenberg DA, Stoy K, Morgan T, Addison CL, Morris SB, Glass M, Wilke C, Xue YY, Sitterding S, Kunkel SL, Burdick MD, Strieter RM (1999). Distinct CXC chemokines mediate tumorigenicity of prostate cancer cells. Am J Pathol.

[B42] Dhawan P, Richmond A (2002). Role of CXCL1 in tumorigenesis of melanoma. J Leukoc Biol.

[B43] Haghnegahdar H, Du J, Wang D (2000). The tumorigenic and angiogenic effects of MGSA/GRO proteins in melanoma. J Leukoc Biol.

[B44] Imada A, Ina K, Shimada M, Yokoyama Y, Nishio Y, Yamaguchi T, Ando T, Kusugami K (2001). Coordinate up-regulation of interleukin-8 and growth-related gene product-a is present in the colonic mucosa of inflammatory bowell disease. Scand J Gastroenterol.

[B45] Mitsuyama K, Tsuruta O, Tomiyasu N, Takaki K, Suzuki A, Masuda J, Yamasaki H, Toyonaga A (2006). Increased circulating concentrations of growth-related oncogene (GRO)-α in patients with inflammatory bowel disease. Dig Dis Sci.

